# Chemical composition of tetraploid *Gynostemma pentaphyllum* gypenosides and their suppression on inflammatory response by NF‐κB/MAPKs/AP‐1 signaling pathways

**DOI:** 10.1002/fsn3.1407

**Published:** 2020-01-14

**Authors:** Bo Wang, Ming Li, Hang Gao, Xiangjun Sun, Boyan Gao, Yaqiong Zhang, Liangli Yu

**Affiliations:** ^1^ Department of Food Science & Engineering School of Agriculture and Biology Shanghai Jiao Tong University Shanghai China; ^2^ Department of Nutrition and Food Science University of Maryland College Park MD USA

**Keywords:** anti‐inflammatory, activator protein 1, *Gynostemma pentaphyllum*, gypenosides, mitogen‐activated protein kinase, nuclear factor‐κB

## Abstract

The chemical composition and anti‐inflammatory activity of gypenosides isolated from tetraploid *Gynostemma pentaphyllum* (GP) leaves were investigated. The gypenosides accounted for 7.43 mg/g of the tested GP sample, which were composed of four major saponins including isomers of gypenoside 1 and 2 (C_47_H_76_O_18_), 3 (C_47_H_76_O_17_), and 4 (C_46_H_74_O_17_). Pretreatment of gypenosides reduced mRNA expressions of the proinflammatory mediators in LPS‐stimulated RAW264.7 macrophage cells, such as IL‐6, IL‐1β, COX‐2, and TNF‐α in a dose‐dependent manner. The secreted protein levels of IL‐6 and TNF‐α, and NO production were also decreased by gypenosides within the concentration range of 50–200 μg/ml. Moreover, the mechanism studies demonstrated that gypenosides (200 μg/ml) treatment significantly inhibited the nuclear translocation of nuclear factor‐κB and activator protein 1 (c‐Fos and c‐Jun) through down‐regulating the phosphorylation of their upstream IκB kinase and mitogen‐activated protein kinases (MAPKs), especially that of c‐Jun N‐terminal kinase and extracellular regulated protein kinase(JNK and ERK), but not that of the p38 MAPK. These results suggested that the gypenosides might have potential anti‐inflammatory effect and use for improving human health.

## Graphical abstract

Our results suggested that the gypenosides might have potential anti‐inflammatory effect and use for improving human health.

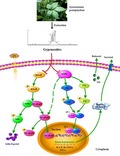

## 1 INTRODUCTION

Inflammation is a natural protective mechanism against various foreign stimuli or physical damage (Kolaczkowska & Kubes, 2013; Wang et al., 2017). As the primary phagocytic cells, macrophages have been considered as the major immune cells exposed to foreign stimuli and initiate the proinflammatory responses through producing inflammatory mediators, such as nitric oxide (NO), tumor necrosis factor (TNF)‐α, interleukin (IL)‐1β, IL‐6, and cyclooxygenase (COX)‐2 (Baatar, Siddiqi, Im, Khaliq, & Hwang, 2018). However, an excessive production of inflammatory mediators is regarded as the main cause for the development of some human diseases, including autoimmune diseases (Kaur, Singh, & Silakari, 2013), type 2 diabetes (Szpigel et al., 2018), hyperlipidemia (Tuzcu, Orhan, Sahin, Juturu, & Sahin, 2017), heart disease (Bartekova, Radosinska, Jelemensky, & Dhalla, 2018), and cancer (Maiuri & O'Hagan, 2018).

Recently, the mitogen‐activated protein kinases (MAPKs) pathway has been recognized as the classical signaling pathway in regulating the inflammatory responses (Kim, Ahn, & Je, 2016). Phosphorylation of MAPKs induces the activation of various transcription factors such as nuclear factor (NF)‐κB and activator protein 1 (AP‐1) and subsequently the production of several inflammatory mediators in activated macrophages. For example, a previous literature showed that ligustilide, a primary volatile essential oil ingredient of Angelica tenuissima, effectively suppressed the expression of inflammatory cytokines by regulating the NF‐κB and MAPK signal pathways (Chung et al., 2012). Proanthocyanidin‐rich red rice extract inhibited the production of NO, IL‐6, TNF‐α, and COX‐2 in LPS‐treated RAW264.7 cells, which were mediated by the inhibition of AP‐1, NF‐κB activation and the MAPKs signaling pathway (Limtrakul, Yodkeeree, Pitchakarn, & Punfa, 2016).

*Gynostemma pentaphyllum* (Thunb.) Makino (GP) is a natural botanical material widely used in food and dietary supplement in Asian countries, and it has been reported to have several health benefits (Li, Lin, Huang, Xie, & Ma, 2016; Norberg et al., 2004; Shen et al., 2018). Gypenosides, the saponins fraction in GP, are considered to be the primary phytochemicals contributing to the health benefits of GP, especially for its anti‐inflammatory activity. For example, Yu et al. (2016) found that gypenosides could alleviate inflammatory cardiac injury by inhibiting NF‐κB p65 activation via the MAPK signaling pathway in H9c2 cell model. However, most of the previous studies were performed using the commercial gypenosides with little information on their chemical compositions and the sources of gypenosides, such as the genotype and plant part of GP.

Our recent study showed that tetraploid GP leaf was a better source for gypenosides than its diploid counterpart or the whole botanical material (Xie et al., 2012). As a continuation, the present study investigated the anti‐inflammatory activity of the gypenosides and its possible molecular mechanism in LPS‐stimulated RAW264.7 macrophage cells. The gypenosides were extracted and isolated from tetraploid *G. pentaphyllum* leaves, and characterized for their chemical compositions by UPLC‐QTOF‐MS analysis.

## 2 MATERIALS AND METHODS

### 2.1 Materials

The leaves of tetraploid *G. pentaphyllum* were provided by Baicaotang Biotechnology Co. Ltd. HPLC grade methanol and acetonitrile were purchased from VWR International, Inc. Dulbecco's modified eagle medium (DMEM), fetal bovine serum (FBS) and streptomycin/penicillin were purchased from GIBCO. Lipopolysaccharides (LPS) from *Escherichia coli*, [3‐(4,5‐dimethylthiazol‐2yl)‐2,5‐diphenyltetrazolium bromide] (MTT), dimethyl sulfoxide (DMSO), protease inhibitor cocktail, Triton X‐100 and 4′, 6‐diamidino‐2‐phenylindole (DAPI) were obtained from Sigma‐Aldrich. Rabbit monoclonal antibodies against IKKα, p‐IKKα/β (ser176/ser180), IκBα, p‐IκBα (Ser32), NF‐κB p65, p‐NF‐κB p65 (Ser536), JNK, p‐JNK (Thr183/Tyr185), ERK, p‐ERK (Thr202/Tyr204), p38 MAPK, p‐p38 MAPK (Thr180/Tyr182), c‐Jun, c‐Fos, PARP, and β‐actin were purchased from Cell Signaling Technology. TRIzol reagent and secondary antibodies conjugated with horseradish peroxidase were purchased from Life Technologies. Phosphatase inhibitors (Roche Diagnostics) were obtained from Beyotime Institute of Biotechnology. All other chemicals and solvents were of analytical grade and used without further purification. Water purified with a Milli‐Q system with resistivity of 18.2 mΩ was used for all experiments.

### 2.2 Extraction and isolation of gypenosides

The crude extract of GP was first obtained following a previous laboratory procedure with some slight modifications (Liu et al., 2015). Briefly, 1.5 kg tetraploid GP dry leaves were reflux extracted with 4 L of slightly boiling 95% ethanol for three times (3, 2, and 1 hr for each, respectively). The combined 95% ethanol extract was suspended in deionized water and extracted sequentially with petroleum ether, ethyl acetate and n‐butanol under reduced pressure. Then, the n‐butanol fractions of dark brown residues were separated with a D‐101 macroporous resin column by successively elution with 20%, 40%, and 60% ethanol. The 60% ethanol fraction was collected, concentrated, and freeze‐dried to obtain dry powders.

The dry powders of GP crude extract were dissolved in methanol and filtered through a 0.22 µm syringe filter (Whatman). The obtained clear yellow solution was injected into a semi‐preparative HPLC with Agilent Zorbax Eclipse XDB‐C_18_ column (250 mm × 9.4 mm, i.d., 5 μm), which was operated at 40°C with a flow rate of 4 ml/min. Acetonitrile (A) and H_2_O (B) were used as the mobile phases, and the system was subjected to the following gradient elution process: 0–6 min, 35%–37% A; 6–18 min, 37%‐42% A; 18–20 min, 42%–35% A. The eluted compounds were monitored at the wavelength of 205 and 254 nm, and the corresponding chromatograms were shown in Figure S1. Based on the chromatograms, the gypenosides fraction was collected from 9.31 to 13.65 min and freeze‐dried as white powders.

### 2.3 Chemical composition of gypenosides by UPLC‐QTOF‐MS analysis

The chemical profile of gypenosides was examined using a Waters Ultra‐performance liquid chromatography (UPLC) coupled with Xevo G2 quadrupole time‐of‐flight (QTOF) mass spectrometer. The UPLC analysis was performed using an Acquity HPLC BEH C_18_ column (100 mm × 2.1 mm, i.d., 1.7 μm) at 40°C. The elution gradient (solution A, water; solution B, acetonitrile) was used as follows: starting at 20% B for 1 min, increased via linear gradient to 90% B at 14 min, and maintained 90% B from 14–16 min. The flow rate was 0.4 ml/min with an injection volume of 10 μl. Mass data were obtained by electro‐spray ionization in negative ion mode and calibrated using the lock‐mass function with leucine encephalin (m/z 556.2771). MS conditions were as follows: capillary voltages, 2.8 kV; sampling cone voltages, 55.0 V; collision energy, 50 eV; source temperature, 100°C; desolvation gas flow, 500.0 L/hr; cone gas flow, 50.0 L/hr; scan range m/z, 100–1,500; scan time, 0.3 s; and inter‐scan time, 0.02 s. Data were collected and analyzed with Waters MassLynx v4.1 software.

### 2.4 Cell culture

The murine RAW264.7 macrophage cells were purchased from the Type Culture Collection of the Chinese Academy of Sciences and cultivated in DMEM supplemented with 10% FBS and 1% penicillin‐streptomycin at 37°C in a 5% CO_2_ incubator (HF90, HealForce Bio‐meditech Holding Corp. Ltd.). The medium was changed every day, and the cells were subcultured after reaching 80%–90% confluence.

### 2.5 Cell viability assay

The MTT assay was used to determine the cell viability in this study (Zheng et al., 2016). In brief, RAW264.7 macrophage cells were seeded in 96‐well plates at a seeding density of 2 × 10^5^ cells/ml and incubated for overnight at 37°C to allow cell attachment. Then cells were treated with different concentrations of extracted gypenosides (0–250 μg/ml) dissolved in DMSO with the final concentration of DMSO in the DMEM medium of 0.1% (v/v). Following 24 hr incubation, the medium was removed and the cells were washed with PBS three times. A volume of 100 μl of DMEM medium containing MTT (0.5 mg/ml) was added into each well and incubated for 4 hr at 37°C. Lastly, the supernatant was removed and 150 μl of DMSO was added. The 96‐well plates were then analyzed with a TECAN Infinite M200 PRO (Tecan Group Ltd.) for absorption at 490 nm. The cell viability was calculated according to the equation below:

$$\text{Cell viability}\left(\%\right)=A_{t}/A_{c}\times 100\%$$

*A*_t_ and *A*_c_ are the absorbance of the gypenosides‐treated groups and blank group cells, respectively.

### 2.6 RNA extraction and RT‐PCR analysis

RAW264.7 macrophage cells were seeded at a density of 2 × 10^5^ cells/ml in 24‐well plates and incubated for 24 hr at 37°C to reach the confluence of 80%. The cells were divided into blank, LPS (1 μg/ml) and LPS + extracted gypenosides (50, 100, 150, and 200 μg/ml) groups. Blank group was treated with DMEM only; LPS group was treated with LPS only; LPS + extracted gypenosides groups were pretreated with different concentrations of gypenosides for 1 hr and then stimulated with LPS (1 μg/ml) for 4 hr. RNA isolation and quantitative real‐time polymerase chain reaction (qRT‐PCR) were conducted according to a laboratory protocol (Zhang et al., 2019). Specific forward and reverse primer sequences used in this study were shown as follows: IL‐6 (Forward: 5′‐CACGGCCTTCCCTACTTCAC‐3′, Reverse: 5′‐TGCAAGTGCATCATCGTTGT‐3′); IL‐1β (Forward: 5′‐GTTGACGGACCCCAAAAGAT‐3′, Reverse: 5′‐CCTCATCCTGGAAGGTCCAC‐3′); TNF‐α (Forward: 5′‐CGAGTGACAAGCCTGTAGC‐3′, Reverse: 5′‐GGTGTGGGTGAGGAGCACAT‐3′); COX‐2 (Forward: 5′ ‐GGGAGTCTGGAACATTGTGAA‐3′, Reverse: 5′ ‐GCACGTTGATTGTAGGTGGACTGT‐3′); and β‐actin (Forward: 5′‐GGAATGGGTCAGAAGGACTC‐3′, Reverse: 5′‐CATGTCGTCCCAGTTGGTAA‐3′).

### 2.7 Cytokines quantification and analysis of NO production

RAW264.7 macrophage cells were treated by the procedure described in Section 2.6. After the treatment, culture supernatant was collected to determine the levels of IL‐6, TNF‐α, and NO production using the commercial mouse kits (eBioscience).

### 2.8 Western‐blotting analysis

RAW264.7 macrophage cells were seeded at a density of 2 × 10^5^ cells/ml into 6‐well plates overnight. Then, the cells were pretreated in the absence or presence of different concentrations of extracted gypenosides (100 or 200 μg/ml) for 1 hr and then stimulated with or without LPS (1 μg/ml) for another 4 hr. After the incubation, RAW264.7 macrophage cells were collected and lysed with 300 μl ice‐cold radioimmunoprecipitation assay (RIPA) buffer containing a protease inhibitor cocktail and phosphatase inhibitors. The whole‐cell lysates were centrifuged at 14,000 *g* for 20 min at 4°C to remove the insoluble materials. Cytoplasmic and nuclear proteins were isolated separately using different extraction kits (Beyotime Biotech). Protein samples were subjected to Western‐blotting analysis according to a previously reported laboratory protocol (Yang et al., 2018).

### 2.9 Immunofluorescence

RAW264.7 macrophage cells were seeded on cover glass‐bottom dishes (Life Sciences) and pretreated in the absence or the presence of extracted gypenosides (200 μg/ml) for 1 hr and then stimulated with or without LPS (1 μg/ml) for 4 hr. Following the incubation, the cells were washed with PBS, fixed with cold 4% paraformaldehyde for 60 min and incubated with the anti‐NF‐κB p65 primary antibody (dilution 1:2,000) at 4°C overnight. Following the reaction, the cells were washed with PBS, treated with Alexa Fluor^®^ 488 conjugate for 1 hr and then stained using DAPI (4 ng/ml) for 60 min at room temperature. After that, the cells were washed with PBS and Prolong Gold Anti‐fade Reagent^®^ (Thermo Fisher Scientific, Inc.) was added to the slide. Lastly, the cells were visualized using a TCS SP8 confocal laser scanning microscopy (Leica Microsystems Inc.).

### 2.10 Statistical analysis

Data were reported as the mean ± standard deviation (*SD*) for three or six replicates determinations. One‐way ANOVA and Tukey's tests were employed to identify differences in means. Statistics were analyzed using the SPSS for Windows (version rel. 10.0.5, 1999, SPSS Inc.). Statistical significance was declared at *p* < .05 or *p* < .01.

## 3 RESULTS AND DISCUSSION

### 3.1 Chemical composition of gypenosides

**Figure 1:**
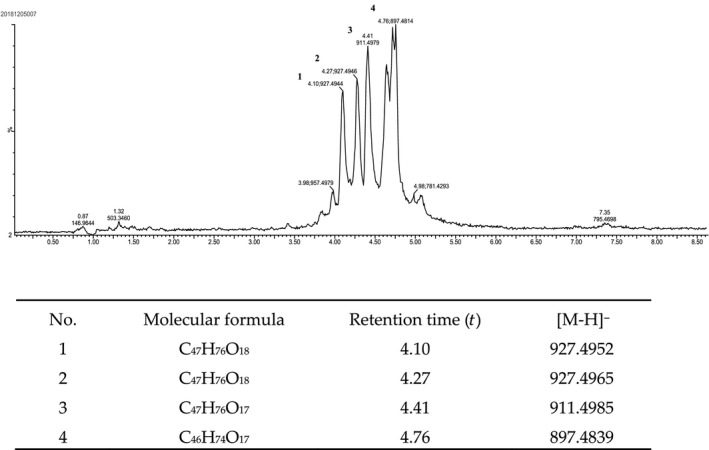
LC‐MS of gypenosides extracted from the tetraploid GP

**Figure 2:**
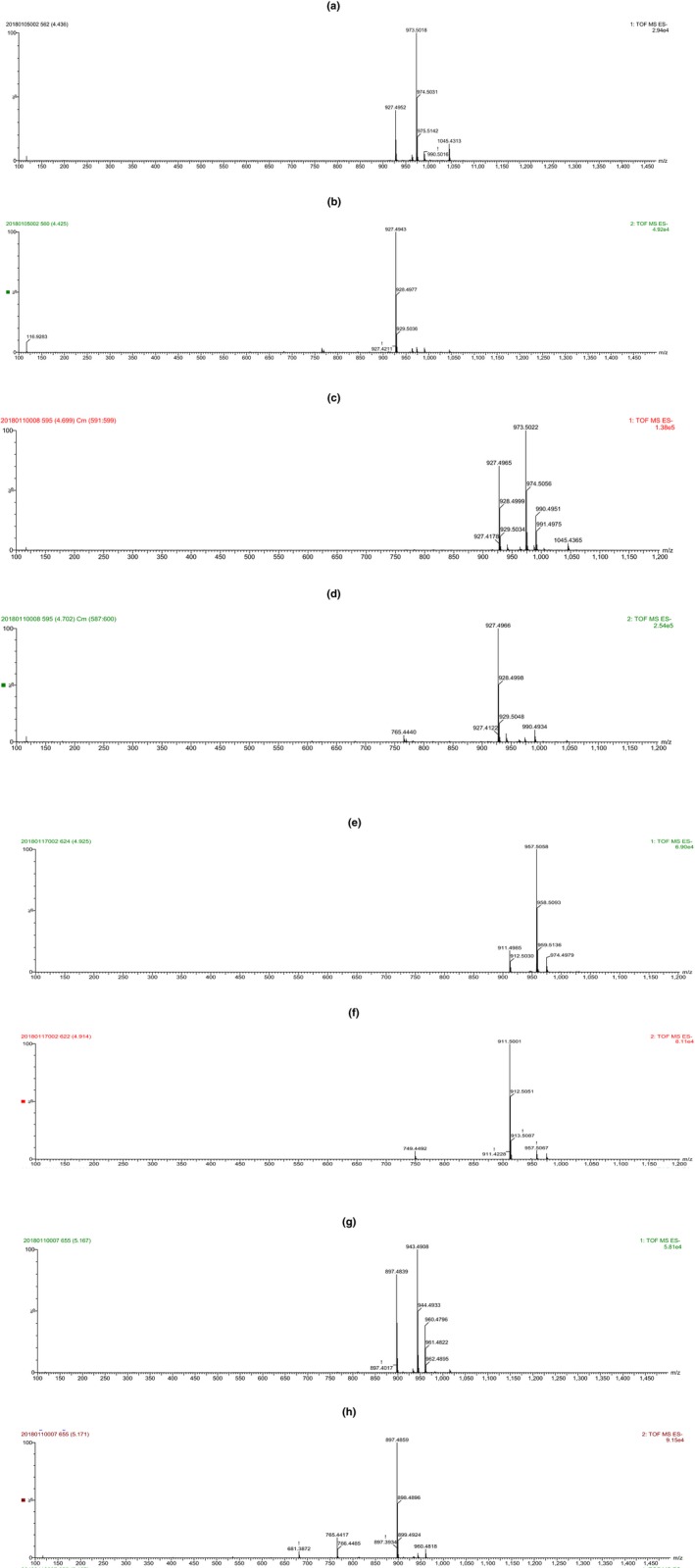
MS and MS‐MS analyses for Gp1 (a, b), Gp2 (c, d), Gp3 (e, f), and Gp4 (g, h)

The gypenosides isolated from the GP crude extract accounted for 7.43 mg/g of the tested GP leaf sample, and their chemical compositions were elucidated by UPLC‐QTOF‐MS analysis. As shown in Figure 1, four major peaks representing four primary saponin components were detected and marked with gypenoside 1 (Gp1), 2 (Gp2), 3 (Gp3), and 4 (Gp4). Besides, their relative area percentages were calculated as 14.95%, 15.28%, 20.02%, and 44.84% of the total peak area, respectively. Gp1 was obtained as the white powder with the quasi‐molecular ion [M‐H]^−^ of 927.4952 (Figure 2a,b), so the molecular formula could be calculated as C_47_H_76_O_18_. Gp2 was also obtained as the white powder. The molecular formula was inferred as C_47_H_76_O_18_, which was same as that of Gp1. Further analysis of the MS and MS‐MS data of Gp1 and Gp2 confirmed that they were isomers (Figure 2a–d). Gp3 was obtained as the light yellow amorphous solid. The quasi‐molecular ion [M‐H]^−^ at 911.4985 indicated that its molecular formula was C_47_H_76_O_17_ (Figure 2e,f). Gp4, a component with the highest content in gypenosides, was obtained as the light yellow powder. The molecular formula was inferred as C_46_H_74_O_17_, which showed a quasi‐molecular ion [M‐H]^−^ at 897.4839 (Figure 2g,h). These four saponins were reported in tetraploid GP but with different relative concentrations, according to the UPLC‐MS data of the four saponin components and our previous reports (Liu et al., 2015, 2016; Yang, Shi, Zhang, Yang, et al., 2013; Yang, Shi, Zhang, & Yu, 2013).

### 3.2 Effect of gypenosides on the cell viability of RAW264.7 macrophage cells

Compared to that of the blank group, there were no significant differences in RAW264.7 macrophage cell viabilities for the gypenosides‐treated groups at the gypenosides concentration range of 50–200 μg/ml (*p* > .05) (Figure S2). Besides, all the cell viabilities were above 99%, indicating the negligible cytotoxicity of gypenosides within these tested concentrations. However, when the concentration of gypenosides increased to 250 μg/ml, a significantly reduced cellular viability of 62.75% was observed (*p* < .01). Therefore, gypenosides at the concentration range of 50–200 μg/ml were selected for the following experiments in this study.

### 3.3 Effect of gypenosides on the mRNA expression of proinflammatory cytokines in LPS‐stimulated RAW264.7 macrophage cells

Several critical proinflammatory cytokines, including IL‐6, IL‐1β, COX‐2, and TNF‐α, are involved in multiple inflammatory pathways, and the inhibition of their mRNA expressions may lead to alleviation of the inflammatory responses (Ogawa et al., 2018). In this study, the effect of gypenosides on the mRNA expressions of IL‐6, IL‐1β, COX‐2, and TNF‐α in LPS‐stimulated RAW264.7 macrophage cells was examined for their potential anti‐inflammatory activities.

**Figure 3:**
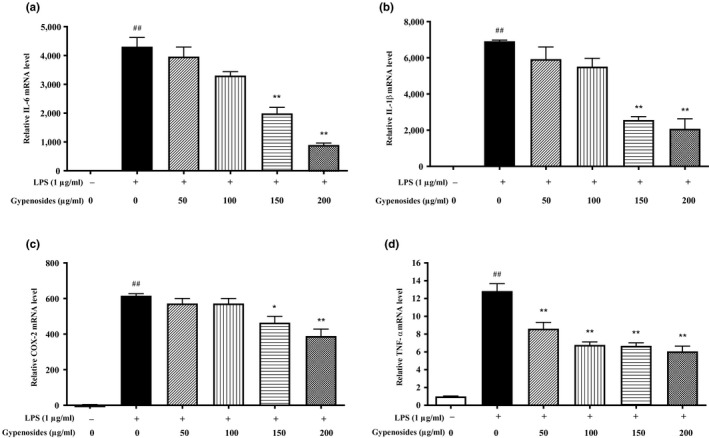
Effects of gypenosides on the mRNA expressions of IL‐6 (a), IL‐1β (b), COX‐2 (c), and TNF‐α (d) in RAW264.7 macrophage cells. LPS stands for lipopolysaccharide. Values are referred as mean ± *SD* and the vertical bars represent the *SD* of six replicates (*n* = 6). *p < .05 and **p < .01 versus the LPS‐treated group. ^##^ *p* < .01 versus the blank group

Compared to the blank, LPS induced significant increases of mRNA expressions of IL‐6, IL‐1β, COX‐2, and TNF‐α in the RAW264.7 macrophage cells (*p* < .01), indicating the successful establishment of the inflammatory model. The gypenosides effectively inhibited the mRNA expressions of IL‐6, IL‐1β, and COX‐2 cytokines in a dose‐dependent manner (Figure 3a–c). A higher treatment concentration of gypenosides was associated with a stronger inhibitory effect on mRNA expressions of cytokines. A significant inhibitory effect for IL‐6, IL‐1β, or COX‐2 was detected when the concentration of gypenosides increased to 150 or 200 μg/ml. Moreover, compared with the LPS‐treated group, the gypenosides‐treated group at the concentration of 50 μg/ml had already significantly inhibited mRNA expression of TNF‐α by about 39% (*p* < .01). Further, increasing the concentration of gypenosides to 100, 150, and 200 μg/ml resulted in 47.20%, 47.96%, and 52.79% inhibition, respectively (Figure 3d).

### 3.4 Effect of gypenosides on the secretion of proinflammatory cytokines in LPS‐stimulated RAW264.7 macrophage cells

**Figure 4:**
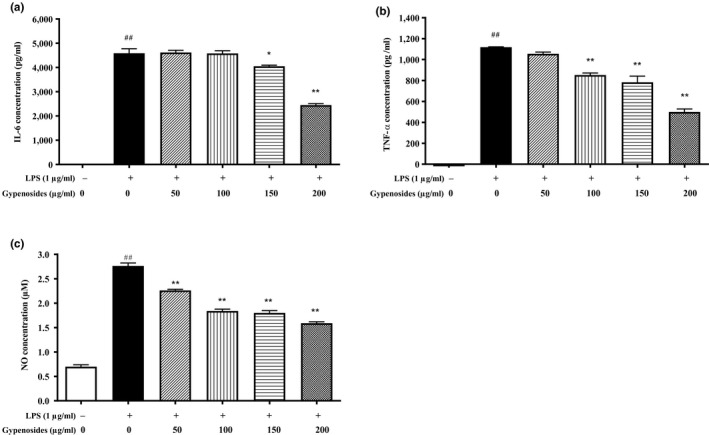
Effects of gypenosides on the secreted protein levels of IL‐6 (a) and TNF‐α (b) and NO production (c) in RAW264.7 macrophage cells. LPS stands for lipopolysaccharide. Values are referred as mean ± *SD* and the vertical bars represent the *SD* of six replicates (*n* = 6). *p < .05 and **p < .01 versus the LPS‐treated group. ^##^ *p* < .01 versus the blank group

To further investigate the anti‐inflammatory activity of gypenosides, the secreted protein levels of IL‐6 and TNF‐α were measured in the medium of LPS‐stimulated RAW264.7 macrophage cells. As shown in Figure 4a,b, both the protein levels of IL‐6 and TNF‐α were significantly increased following the LPS stimulation (*p* < .01) and pretreatment of the gypenosides inhibited the secretion of IL‐6 and TNF‐α in the culture medium. A significant inhibitory effect for IL‐6 was observed at the gypenosides concentration of 150 μg/ml (*p* < .05) and 200 μg/ml (*p* < .01) (Figure 4a), while a significant inhibition for TNF‐α was found within the gypenosides concentration range of 100–200 μg/ml (*p* < .01) (Figure 4b). Changes in the protein levels of IL‐6 and TNF‐α were consistent with those observed in their mRNA expression levels (Figure 3a,d).

Furthermore, the intracellular nitric oxide (NO) release is also involved in the signal transduction of inflammatory responses (Sagar et al., 2017). It is important to inhibit the over‐production of NO in response to inflammatory stimuli, which can induce proinflammatory responses in inflammatory disorders (Lively & Schlichter, 2018). Compared to the blank, the NO level was significantly increased in LPS‐stimulated RAW264.7 macrophage cells (*p* < .01) (Figure 4c), and the pretreatment of gypenosides inhibited the NO production in a dose‐dependent manner. Significant differences were observed at all the tested gypenosides concentrations (50–200 μg/ml) (*p* < .01).

Overall, gypenosides could reduce the secretion levels of IL‐6 and TNF‐α and NO production in LPS‐stimulated RAW264.7 macrophage cells. These observations were consistent with a previous literature that saponins from ginseng and panax japonicus suppressed the protein levels of some proinflammatory cytokines, such as TNF‐α, COX‐2, IL‐1β, and IL‐6 (Baek et al., 2016).

### 3.5 Gypenosides suppressed LPS‐stimulated NF‐κB activation in RAW264.7 macrophage cells

**Figure 5:**
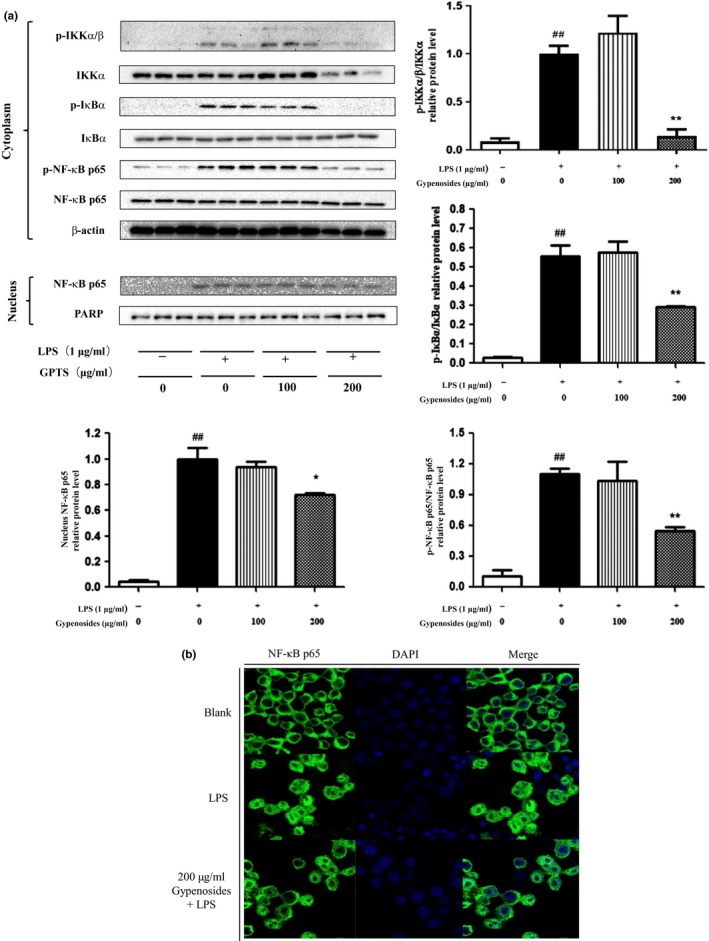
Effects of gypenosides on the protein levels of IKKα, p‐IKKα/β, IκBα, p‐IκBα, NF‐κB p65, p‐NF‐κB p65 (a). Immunofluorescence staining and confocal microscopy was used for observing NF‐κB p65 (green) translocation into the nucleus (blue) (b). Values are referred as mean ± *SD* and the vertical bars represent the *SD* of three replicates (*n* = 3). *p < .05 and **p < .01 versus the LPS‐treated group. ^##^ *p* < .01 versus the blank group

Previous studies have showed that NF‐κB is a crucial transcription factor involved in the regulation of proinflammatory cytokines (Jeon et al., 2013; Yang et al., 2017). NF‐κB normally exists within the cytoplasm of unstimulated cells as an inactive complex, which is composed of the p65 subunit bound to the inhibitory proteins of the IκBα family. When cells are stimulated by LPS, the IκB kinase complex (IKK), which is an important upstream kinase for phosphorylation of IκBα and subsequent IκBα degradation in macrophages is activated. This pathway allows the translocation of unbound NF‐κB p65 into the nucleus to trigger the transcription of downstream proinflammatory cytokines (Noort et al., 2014). An earlier study showed that soy saponins could reduce inflammation response by suppressing NF‐κB activation in macrophages (Zha et al., 2014). Therefore, it is interesting whether and how gypenosides may alter NF‐κB pathway to have their anti‐inflammatory activity in LPS‐stimulated RAW264.7 macrophage cells. As shown in Figure 5a, LPS alone induced a significant increase in both IKKα/β and IκBα phosphorylation of RAW264.7 cells (*p* < .01), while this effect was significantly suppressed by gypenosides at the concentration of 200 μg/ml (*p* < .01). Furthermore, compared to those of the blank group, cells treated with LPS alone resulted in a significant increase in the phosphorylation of NF‐κB p65 in the cytosol and nuclear translocation of NF‐κB p65 (*p* < .01). Pretreatment with gypenosides (200 μg/ml) also reversed this effect and resulted in 50.37% and 27.91% of inhibition, respectively (*p* < .01 for cytosolic p‐NF‐κB p65, *p* < .05 for nucleus NF‐κB p65). However, gypenosides at the concentration of 100 μg/ml did not show a significant suppression effect on NF‐κB p65 activation in LPS‐stimulated RAW264.7 macrophage cells.

To further confirm whether pretreatment with gypenosides (200 μg/ml) could inhibit NF‐κB p65 nuclear translocation, the immunofluorescence assay was performed to support the Western‐blotting results. It was found that NF‐κB p65 (denoted by green fluorescence) was localized in the cytosol for the blank group (Figure 5b). After LPS stimulation, NF‐κB p65 proteins translocated to the nucleus, but it was effectively inhibited by treating with gypenosides. Taken together, these results suggest that gypenosides could suppress LPS‐stimulated inflammatory responses by inhibiting IKK/NF‐κB activation in RAW264.7 macrophages.

### 3.6 Gypenosides suppressed LPS‐stimulated MAPKs/AP‐1 activation in RAW264.7 macrophage cells

**Figure 6:**
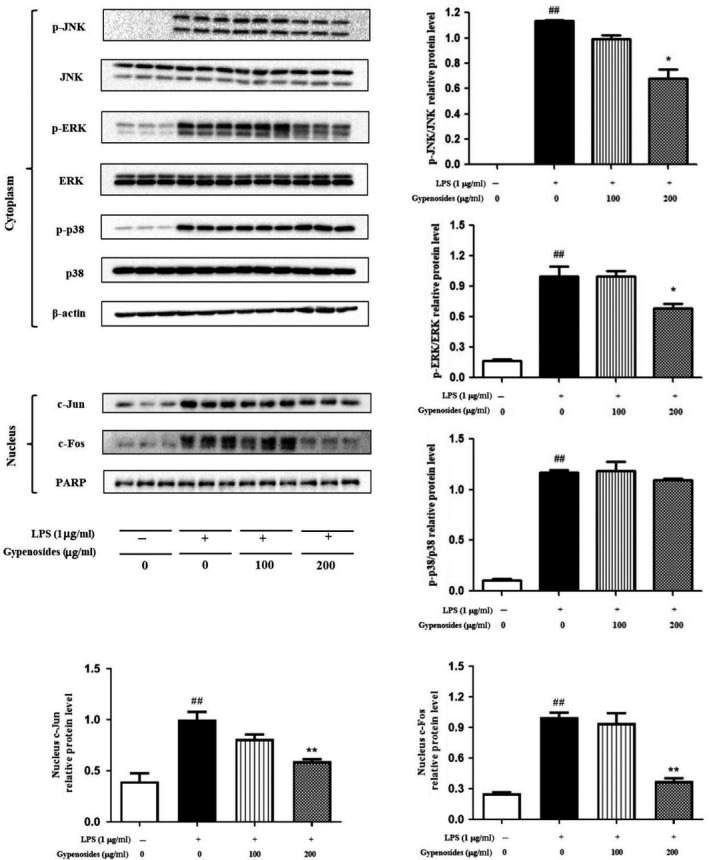
Effects of gypenosides on the protein levels of JNK, p‐JNK, ERK, p‐ERK, p38, p‐p38, c‐Jun, and c‐Fos. Values are referred as mean ± *SD* and the vertical bars represent the *SD* of three replicates (*n* = 3). *p < .05 and **p < .01 versus the LPS‐treated group. ^##^ *p* < .01 versus the blank group

The MAPKs, including JNK, ERK, and p38 MAPK signaling pathways, are considered the classical pathways that regulate the inflammatory response (Limtrakul et al., 2016). It was previously reported that the inhibition of MAPKs pathway was sufficient to block the proinflammatory mediators in macrophages by the LPS induction (Kim et al., 2018; Zhu et al., 2016). In order to explore whether MAPKs could also be affected by gypenosides in LPS‐stimulated RAW264.7 macrophages, cells were pretreated with gypenosides prior to LPS stimulation and the phosphorylation of JNK, ERK, and p38 MAPKs was also analyzed by Western‐blotting analysis. As shown in Figure 6, gypenosides showed no effect on the total expression level of JNK, ERK, and p38, but it specifically decreased the expression level of phosphorylated JNK and ERK. Compared to those of the LPS‐only treated group, gypenosides (200 μg/ml) significantly decreased the phosphorylation of JNK and ERK by 40.15% and 31.71%, respectively (*p* < .05), whereas gypenosides at the concentration of 100 μg/ml did not appear to have the obvious suppression effect (*p* > .05). Interestingly, the phosphorylation level of p38 was not affected by gypenosides even at the concentration of 200 μg/ml. Our findings were consistent with an earlier report that JNK and ERK but not the p38 pathway was involved in the inflammatory inhibition of fructus sophorae on LPS‐stimulated RAW264.7 macrophage cells (Choi & Kang, 2016).

In addition, some previous studies have shown that the phosphorylation of MAPKs, especially for JNK and ERK, could further trigger the activity of its downstream AP‐1 (Chun et al., 2019; Kang, Hong, Kang, Park, & Choi, 2015). AP‐1, a heterodimeric protein complex composed of Jun and Fos families, is a transcription factor that also plays a key role in regulating inflammatory responses (Park & Song, 2013). Since the suppression effect of gypenosides on the phosphorylation of JNK and ERK were observed in this study, we further investigated whether it could also regulate the AP‐1 (c‐Jun and c‐Fos). As shown in Figure 6, gypenosides at the concentration of 200 μg/ml did significantly inhibited the nuclear translocation of c‐Jun and c‐Fos compared with the LPS‐only treated group in RAW264.7 macrophage cells, which showed 40.59% and 62.48% of inhibitory ratio, respectively (*p* < .01). For the gypenosides at the concentration of 100 μg/ml, no statistically significant suppression effect was observed (*p* > .05), which was consistent with that observed on the phosphorylation of JNK and ERK. All these findings demonstrated that the anti‐inflammatory effect of gypenosides may also be mediated by decreasing the LPS‐stimulated nuclear translocation of AP‐1 though inhibiting the phosphorylation of JNK and ERK.

## 4 CONCLUSION

In summary, gypenosides containing four major saponins from tetraploid *G. pentaphyllum* leaves could inhibit the expression and secretion of inflammatory mediators IL‐6, IL‐1β, COX‐2, TNF‐α, and NO in LPS‐stimulated RAW264.7 macrophage cells. Furthermore, the possible mechanism for this effect involves the suppression of NF‐κB and AP‐1 nuclear translocation through down‐regulating the activity of their upstream IKK, JNK, and ERK. These findings suggest the potential utilization of tetraploid *G. pentaphyllum* leaves or its gypenosides in functional food and dietary supplements to improve human health.

## CONFLICT OF INTEREST

The authors declare that there is no conflict of interests.

## ETHICAL APPROVAL

This article does not involve any human or animal studies.
