# Supplementary material for: Chemical composition of tetraploid Gynostemma pentaphyllum gypenosides and their suppression on inflammatory response by NF‐κB/MAPKs/AP‐1 signaling pathways
Source: Food Sci Nutr. 2020 Jan 14;8(2):1197–207. doi: 10.1002/fsn3.1407 (PMC7020333; doi:10.1002/fsn3.1407)
Supplement: Supplementary file 1 [file FSN3-8-1197-s001.docx]

Chemical composition of tetraploid *Gynostemma pentaphyllum* gypenosides and their suppression on inflammatory response by NF-κB/MAPKs/AP-1 signaling pathways

Bo Wang^1^, Hang Gao^1^, Ming Li^1^, Xiangjun Sun^1^, Boyan Gao^1^, Yaqiong Zhang^1,^*, Liangli (Lucy) Yu^2^

^1^Department of Food Science & Engineering, School of Agriculture and Biology, Shanghai Jiao Tong University, Shanghai 200240, China

^2^Department of Nutrition and Food Science, University of Maryland, College Park, MD 20742, United States

* To whom correspondence should be addressed:

Yaqiong Zhang, Ph. D.

Tel.: 021-34204538; E-mail: [yqzhang2006@sjtu.edu.cn](mailto:%20yqzhang2006@sjtu.edu.cn)

(A)


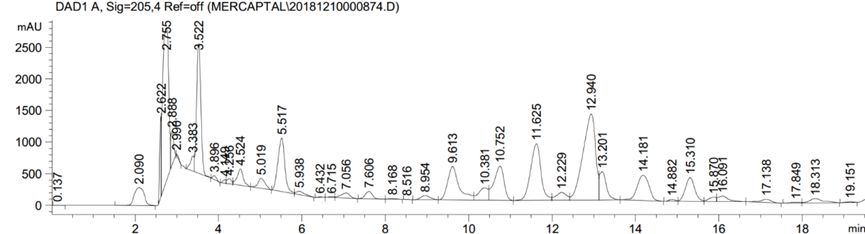


(B)


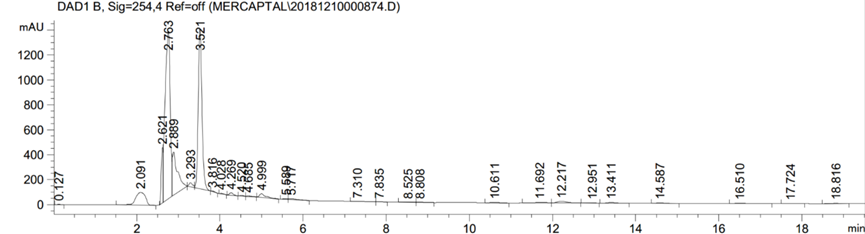


Figure S1. Chromatograms of crude extracts of tetraploid gypenosides detected by HPLC-DAD at 205 nm (A) and 254 nm (B).

Figure S2. Cell viability of RAW264.7 macrophage cells after treatment with different concentrations of gypenosides. Values are referred as mean ± SD and the vertical bars represent the SD of six replicates (n=6). ** *p* < 0.01 vs the blank group.
